# Special Issue on “Enzymes as Biocatalysts: Current Research Trends and Applications”

**DOI:** 10.3390/ijms232416209

**Published:** 2022-12-19

**Authors:** Sérgio F. Sousa

**Affiliations:** 1 Associate Laboratory i4HB—Institute for Health and Bioeconomy, Faculty of Medicine, University of Porto, 4200-319 Porto, Portugal; sergiofsousa@med.up.pt; 2 UCIBIO—Applied Molecular Biosciences Unit, BioSIM—Department of Biomedicine, Faculty of Medicine, University of Porto, 4200-319 Porto, Portugal

Enzymes are able to catalyze a wide diversity of chemical reactions in nature, and they do it at an amazing level. In fact, without the action of enzymes, many of the reactions of biochemical relevance would be so slow that they would not take place under the mild conditions of temperature and pressure that characterize life. Enzymes enable these reactions by accelerating their rates of occurrence, in many cases by over a million times. In addition, they offer a variety of benefits that no other synthetic catalyst can normally offer: high turnover, regio- and stereoselectivity, and the ability to react in environmentally friendly manufacturing processes. For these reasons, the application of enzymes as biocatalysts in industrial applications has been gaining increasing relevance for a variety of industries and applications.

This Special Issue focuses on the use of enzymes as biocatalysts and includes five original articles and four review papers describing aspects and methods related to their development and application.

Bulygin et al. [1] present a study on the mechanisms of damage recognition and catalysis by Apurinic/apyrimidinic (AP) endonucleases, key DNA repair enzymes in the base excision repair (BER) pathway. The authors focus on four homologous APE1-like endonucleases, namely insect (*Drosophila melanogaster*) Rrp1, amphibian (*Xenopus laevis*) APE1 (xAPE1), fish (*Danio rerio*) APE1 (zAPE1), and human APE1 (hAPE1). These enzymes were studied by atomistic molecular dynamics simulations, enabling a comparison of the data obtained from these simulations with their known catalytic efficiency to obtain insight into the differences in the cleaving efficiency of different damaged nucleotides. The results obtained highlight the importance of the amino acid residues within a specific loop containing residues Asn222-Ala230 in the formation of the catalytic complex. In addition, the study provides a detailed characterization of the set of interactions at the active-site amino acid residues with the different damaged DNA substrates.

Tohar et al. [2] report the screening of collagenase activity for directed enzyme applications of bacterial lysate. In particular, the authors have modified and validated the application of a previously reported fluorogenic assay using 3,4-dihydroxyphenylacetic acid for the quantitation of collagen and applied it in the detection of bacterial collagenase activity in bacterial lysates. This approach enables the evaluation of collagenase activity in a 96-well deep-well-plate format in addition to the screening of optimized protein variants from a genetic library. The authors have demonstrated that this repurposed assay is highly selective. Furthermore, they have shown that it enables the detection of collagenase activity with collagen in the lysates, thus being suitable for the identification of variants with improved activity.

Herrera et al. [3] describe the rational design of resveratrol O-methyltransferase (OMT) for the bioproduction of pinostilbene, a stilbene that is regarded as a promising alternative to resveratrol, with superior bioavailability. In particular, the authors have engineered a resveratrol OMT from *Vitis vinifera* (VvROMT), the OMT with the highest catalytic efficiency in di-methylating resveratrol, to yield pterostilbene. Following the creation of a 3D model of VvROMT and the application of different computational methods, the authors have identified four critical binding site amino acid residues and have used this information and data on the literature to rationally design new mutants, with improved activity and modified substrate selectivity. The results demonstrate that this enzyme has potential for the tailor-made production of stilbenes.

Rosenbergova et al. [4] describe the optimization of recombinant myrosinase production in *Pichia pastoris*. This enzyme is important in plant defense as it catalyze the hydrolysis of glucosinolates to different volatile compounds, most notably to isothiocyanate. This molecule has been demonstrated to have neuroprotective and chemo-preventive properties, making myrosinase a potentially attractive enzyme for the pharmaceutical industry. In this study, the authors report the high-cell-density cultivation of the *P. pastoris* KM71H (Mut^S^) strain expressing TGG1 myrosinase from *Arabidopsis thaliana* in what is the first report of this type of strain for the expression of myrosinase.

Chang et al. [5] describe an amino-acid-sequence-based algorithm for increasing the heat resistance of a protein while maintaining its functions. The authors have used the adenylate kinase (ADK) family as a model system and were able to identify a series of amino acid sites related to thermostability. This information was then used by the authors to engineer single- and double-point mutants, which were shown to have a higher thermal denaturation temperature while preserving most of the catalytic function at ambient temperatures.

Patti and Sanfilippo [6] present a detailed review on stereoselective and promiscuous reactions catalyzed by lipases. In particular, the authors focus on lipase-catalyzed promiscuous reactions that produce optically active products, offering a current state-of-the-art and presenting a perspective in this field of asymmetric synthesis.

Naeem et al.’s work [7] presents a detailed review on the advanced enzyme molecular engineering approaches currently available and on how these strategies can be used to enhance the thermostability of enzyme breakers in the upstream oil and gas industry.

Magalhães et al. [8] review the current status on the use of enzymatic catalysis for the degradation of plastic PET. In this work, the authors present an overview of currently known PET-degrading enzymes, highlighting the structural and activity characteristics of these enzymes and summarizing recently reported engineering efforts to improve their activity.

Finally, Lyagin and Efremenko [9] present a review focusing on the enzymes that are able to react with organophosphorus compounds as detoxifiers, acting via hydrolysis or oxidation/reduction. In particular, the authors discuss their identity and structural differences, catalytic mechanisms, and applicability and specificity, also highlighting future developments and applications involving their use.

Overall, these nine contributions constitute a diverse collection on the use of enzymes as biocatalysts, presenting several distinct examples on their immense potential and wide applicability—aspects that have been growing drastically in recent years. The future of the use of enzymes as biocatalysts is clearly bright.
